# Improving Primary Healthcare: Applying Grapevine (*Vitis vinifera*) Principles to Lebanon’s Vision 2030

**DOI:** 10.3390/healthcare14172741

**Published:** 2026-08-28

**Authors:** Karim W. Barake, Aaron Kinyu Hoshide, Salim M. Adib, Kimberly Samaha

**Affiliations:** 1 Born Global Foundation, 254 Commercial Street, Portland, ME 04101, USA; barake@bu.edu (K.W.B.); ksamaha@bornglobalfoundation.org (K.S.); 2 Kilachand Honors College, Boston University, Boston, MA 02215, USA; 3 Sensei Economic Solutions, LLC, Rome, GA 30165, USA; 4 AgriSciences, Instituto de Ciências Agrárias e Ambientais, Universidade Federal de Mato Grosso, Sinop 78555-267, MT, Brazil; 5 Department of Epidemiology and Biostatistics, American University of Beirut, P.O. Box 11-0236, Beirut 1107 2020, Lebanon; sa193@aub.edu.lb

**Keywords:** Agent-Based Approach, biomimicry, healthcare policy, Lebanon, Need Score, primary healthcare, soft systems methodology, *Vitis vinifera*

## Abstract

**Highlights:**

**What are the main findings?**

- Lebanon’s primary healthcare system lacks institutionalized sensing, adaptive redistribution, and integrated referral architecture, resulting in persistent misalignment between population health need and service capacity.
- An Agent-Based Approach using a biomimicry-inspired framework inspired by the drought-resistant root system of *Vitis vinifera* can translate these deficiencies into actionable policy mechanisms using standardized Need Scores, need-responsive spatial redistribution, and structurally anchored referral networks.

**What are the implications of the main findings?**

- Primary healthcare reform in Lebanon should shift from discretionary, infrastructure-led expansion toward rule-based need-responsive governance that institutionalizes system sensing, adaptive redistribution, and continuous feedback.
- Biomimicry can offer transferable design logic for health system resilience, demonstrating how biological principles can be operationalized at the policy level to strengthen primary care performance in fragile and crisis-prone settings.

**Abstract:**

**Background/Objectives**: While Lebanon’s Vision 2030 prioritizes strengthening Lebanon’s chronically constrained, fragmented, and shocked primary healthcare system, implementation is hindered by weak sensing mechanisms, misaligned spatial distribution, and poorly integrated referral pathways. This study develops a preliminary, context-specific, systems-level healthcare policy assessment and framework for primary healthcare reform by applying biomimicry principles derived from the drought-resistant root architecture of grapevine (*Vitis vinifera*). **Methods**: Publicly available data from the Lebanese Ministry of Public Health, national assessments, and non-governmental organization registries were synthesized to examine mismatches between healthcare need and service capacity. We developed a preliminary healthcare Need Score for Lebanon’s eight governorates based on clinical demand, capacity strain, and socioeconomic vulnerability. An Agent-Based Approach within a biomimicry framework was used to translate grapevines’ biological strategies into healthcare policy design principles. **Results**: Structural deficiencies were identified in Lebanon’s primary healthcare system, including absence of standardized need-based sensing, uneven geographic service distribution, weak-referral integration, and limited adaptive feedback. Our preliminary Need Score was used four clinical demand indicators for iron deficiency, neonatal and maternal deaths, pre-mature births and birth defects, and disease incidence. Service-capacity strain indicators were primary healthcare center density and staffing. Finally, socioeconomic vulnerability indicators were percentages for non-Lebanese population and food insecurity. The proposed policy framework can translate frontline stress signals to rule-based responses, guide adaptive redistribution of capacity, and structurally anchor primary healthcare centers to referral hubs through defined catchments and electronic referral loops. **Conclusions**: Biomimicry can shed insight into how to improve primary healthcare governance under chronic scarcity. Need Scores and biological resilience principles can strengthen primary healthcare systems in low-resource settings while aligning with healthcare policy objectives like those in Lebanon’s Vision 2030.

## 1. Introduction

After World War I and the collapse of the Ottoman Empire in 1918, the League of Nations gave France an “administrative mandate” over Syria and Lebanon [1,2]. Building on existing Ottoman measures, the French administration introduced bureaucratic and medically-oriented public health structures [3], establishing the Ministry of Hygiene and Public Assistance [4]. While the French aimed to create a centralized state, in practice the established authority-sharing process enabled sectarian lobbying and political patronage when allocating all services, including in health and social welfare [5,6].

The resulting privatized, fragmented, and unequal service model became institutionalized as Lebanon’s Ministry of Health and Social Affairs during the first three decades following Lebanon’s independence in 1943 [1,5]. The Lebanese Civil War (1975–1990) devastated the country’s public health infrastructure leaving Lebanon’s new version of health governance, the Ministry of Public Health (MoPH), almost entirely dependent on the private hospital sector with 80% of its budget going to subsidize care provided for non-insured patients in private facilities. In Lebanon’s 2005 Health Reform Plan strengthening primary care, human resources, and hospital autonomy, the MoPH became an “insurer of last resort” for at least 42% of the population lacking hospitalization coverage [7].

The MoPH also established partnerships for service delivery in primary healthcare (PHC) with non-governmental organizations (NGOs), religious institutions, willing municipalities and political parties [8,9,10,11]. In 1994, the MoPH created a national PHC network to regulate access through all partners’ services to affordable, standardized primary care for vulnerable populations [12,13]. Despite only 5.8% of total government spending being devoted to healthcare, PHC was prioritized in health policy [5,12,13]. By the late 1990s, PHC formed the backbone for public service delivery and included 226 centers by 2020, mostly NGO- or municipality-run, subsidizing ambulatory healthcare for more than 1 million people annually [12].

In 2009, the MoPH introduced national accreditation standards for PHC centers, which set uniform benchmarks for infrastructure, staffing, service delivery, and patient safety [14]. The program strengthened governance, documentation, patient flow, and infection control by assessing infrastructure, service quality, patient rights, and management systems in PHC centers nationally [15]. However, expansion was limited by resource constraints, uneven geographical distribution of resources, and sporadic technical support.

Starting in 2011, the PHC network was strained by the Syrian refugee crisis, where 1+ million refugees increased Lebanon’s population by ~30% [7,16]. The MoPH integrated refugee services into the national system instead of creating parallel structures, thus improving coordination but straining resources. PHC annual utilization grew from 700,000 (2009) to 1.2 million (2014) participants, 47% of whom were Syrian refugees [12,16]. Despite this strain, Lebanon’s healthcare system absorbed shocks without complete collapse, thanks to flexibility in the private sector and MoPH partnerships and support from international agencies, as well as diversification and decentralization [15,16]. Since 2019, Lebanon entered its worst financial crisis in modern history, followed by multiple converging conflicts that pushed Lebanon’s healthcare sector to the brink of collapse [17]. The Lebanese Lira lost over 90% of its value, eroding purchasing power for both households and healthcare institutions [5,18,19]. Hospitals faced skyrocketing import costs for medicines, consumables, and equipment [5,18]. Private hospitals, providing most care, were owed USD 1.3 billion by the government, and out-of-pocket spending soared from 33.1% to over 85% of total health expenditures, making healthcare unaffordable for most [5,17,18,19].

The first COVID-19 case was diagnosed in February 2020 amid the historical bankruptcy of the entire banking system. Lebanon’s hospitals, already struggling with financial shortfalls, were rapidly overwhelmed with resource shortages partially mitigated through the World Health Organization (WHO) and NGO support [20]. The PHC network saw a 70% decline in visits with disruptions to routine care (e.g., maternal health, chronic illness care…). The Beirut Port Explosion on 4 August 2020 destroyed or damaged six major hospitals and shut down dozens of PHC facilities in Beirut, killed over 200 people and injured 6500+, adding immediate trauma care demands. Hospitals exhausted two months’ worth of medical supplies in days, with total economic/physical losses estimated at USD 8 billion [18,20]. PHC funding was cut by the MoPH, causing struggles with basic utilities, rising costs, and unpaid staff, forcing operational compromises [13]. The blast, pandemic, economic collapse, and lack of trust in government created a layered crisis, overwhelming Lebanon’s healthcare system.

**Figure A1:**
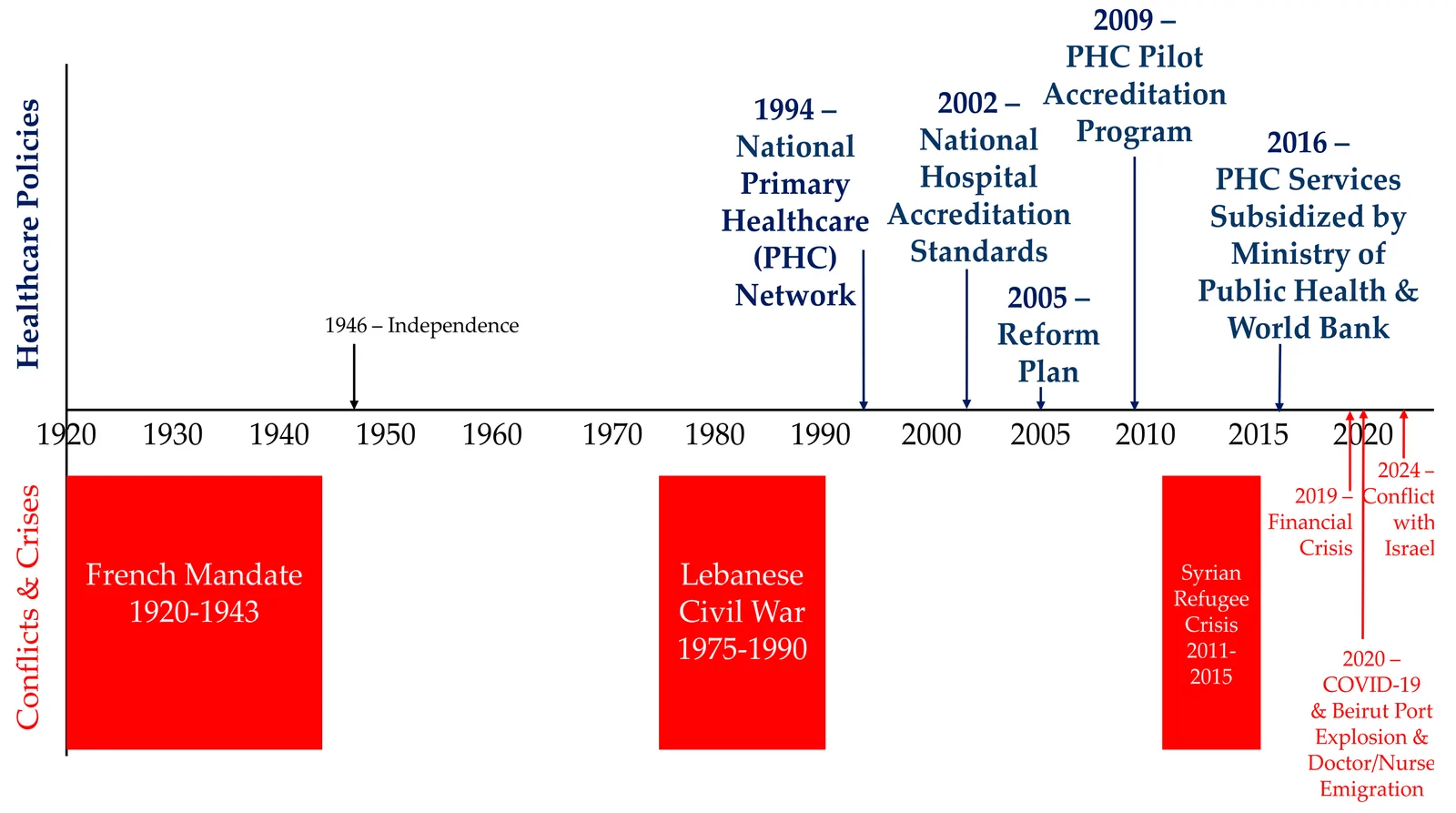
Timeline of major recent healthcare polices and conflicts impacting Lebanon’s primary healthcare system.

Over the next few years, thousands of doctors (~40% of total) and nurses (20%) emigrated due to unpaid wages, burnout, unsafe conditions, better salaries abroad, political instability, and hyperinflation [18,21,22]. Some hospitals reduced services or faced permanent closure, furloughed staff, or became entirely dependent on foreign aid. PHC centers experienced drug and staffing shortages as well as power outages [13]. Private hospitals began demanding upfront payments in USD from uninsured patients and medical tourism from neighboring Arab countries collapsed [21]. Lebanon’s open conflict with Israel, which flared up in 2024, has injured over 17,000 people, and killed 4000+ including at least 241 health workers [22]. Subsequently, 133 PHC centers and eight hospitals closed in combat zones, restricting 30% of private hospital beds. The MoPH has operated at less than 30% staff capacity [22]. Absent a return to peace and profound structural reform, Lebanon’s public health infrastructure risks near-total collapse from continuing crises (Figure A1).

Despite the bleak reality, plans for PHC structural reform were nevertheless launched in January 2023, when the MoPH, with support from the WHO, uncovered “Vision 2030,” the Lebanese National Health Strategy to reform the country’s healthcare system into a more equitable, resilient, and people-centered system by 2030 [23]. Vision 2030 articulates five core directions: improving governance, achieving universal health coverage with financial protection, enhancing health service delivery, strengthening health security and emergency preparedness, and promoting health and disease prevention. Vision 2030 emphasizes primary care, efficient resource use, digital health systems, public-private collaboration, and resource optimization over system expansion. Central to this vision is strengthening PHC as the main entry point to care, reducing unnecessary hospital utilization, and using referral mechanisms to ensure that costly diagnostics and specialist services are accessed only when clinically indicated. If fully implemented, Vision 2030 provides a coherent strategic roadmap for the coming decades.

Lebanon requires a diversified approach to improving its healthcare system. Mulyono et al. (2023) used an Agent-Based Approach (ABA) to propose policy recommendations for Complex Adaptive Systems improvements [24]. In this ABA applied to our analysis, biomimicry inspiration from the common grapevine (*Vitis vinifera*) identified potential areas for such improvements. *Vitis vinifera* is a highly adaptable perennial woody vine with a climbing growth habit used for both winemaking and table consumption. It originated in the Mediterranean region and Central Asia before spreading worldwide, particularly in Mediterranean-type regions where drought and water scarcity are defining agricultural constraints [25,26,27,28,29,30]. *Vitis vinifera* is known for its ability to access water deep in the ground with a root system often extending beyond 6 m in depth with lateral spread exceeding 10 m [27], which allows it to maintain productivity under prolonged drought [28,30]. The grapevine’s architecture is highly plastic, shifting between coarse structural roots and fine laterals depending on water availability, enhancing both anchorage and absorptive efficiency [31,32].

Biomimicry involves applying “Life’s Principles” to study non-biological system functions [33,34]. Biomimicry studies and translates nature’s time-tested strategies into human systems, technologies, and policies that are efficient, resilient, and sustainable [35,36,37,38,39,40]. Nature-inspired strategies have yielded concrete, high-impact innovations in medicine, architecture, textiles, and urban planning, showing that biomimicry is a practical engineering and design method, not purely a conceptual tool [38,41,42,43,44,45]. Biomimicry provides a rigorous framework for redesigning complex systems [46,47], such as national health policy, given environmental and resource constraints [39]. Biomimicry frameworks apply systems-level strategies such as feedback loops, self-regulation, and nested subsystems, enabling national health networks to monitor, adapt, and optimize based on real-time data [42,48,49].

We present an innovative, context-specific health policy framework for “Lebanon’s Vision 2030” which applies an ABA based on biomimicry principles to propose a healthcare system that is resilient, adaptable, and sustainable under conditions of chronic resource scarcity [41]. The specific objectives of this study were three-fold. First, Lebanon’s healthcare infrastructure adequacy was evaluated relative to population (Lebanese and non-Lebanese) at the governorate level. Second, we developed a preliminary healthcare Need Score requiring future validation. Third, functional strategies from nature were identified that can be translated into health system design principles and public policy recommendations to strengthen primary care, enhance resource-allocation, and incorporate evidence-based feedback mechanisms for adaptive management under Vision 2030.

## 2. Materials and Methods

### 2.1. Healthcare Data and Need Score

We analyzed and/or summarized public and private hospital data by governorate and population data by municipality from the MoPH (2010–2019) in Lebanon [50]. We compared MoPH 2017 hospital data to the Hemadeh et al. (2020) survey of PHC centers conducted from December 2017 to January 2018 [12]. We then summarized non-governmental organizations with healthcare centers and mobile clinics [51] by governorate. All NGOs included for comparison to hospitals and PHC centers were checked for establishment prior to 2017. Lebanon’s eight governorates running north to south are Akkar, North, Baalbek-Hermel, Beqaa, Mount Lebanon, Beirut, South, and Nabatieh. We also contrasted subsidized PHC patients treated in hospitals in each municipality versus patients who are residents of each municipality [50] and aggregated to the governorate level.

We specified a PHC Need Score (Supplementary Materials—File S1: Table S1) following the logic of established need-weighted allocation tools inspired by Thailand’s Quality and Outcomes Framework [52], Sweden’s Care Need Index [53,54,55], and England’s need-weighted staffing formulas [56,57]. This Need Score is built as a weighted sum of normalized domain sub-scores, with each domain populated by a small set of high-yield indicators. Indicators for each of the three domains of clinical demand, service-capacity strain, and socioeconomic vulnerability are shown in Supplementary Materials—File S1: Table S2. Domain sub-scores can be normalized using safe and crisis thresholds (Supplementary Materials—File S1: Tables S1 and S2) [58,59,60,61,62]. Each indicator and domain score are on a 0 to 1 scale with the resulting Need Score on a 0 to 100 scale with 0 = least need and 100 = highest need (Supplementary Materials—File S2). Both domain and within-domain weights were set to be equal (Supplementary Materials—File S1: Table S2).

Domain indicators for Lebanon’s Need Score were selected (Table A1). Clinical demand was represented by iron deficiency for children ages 6 to 59 months, defined as plasma ferritin below 12 µg/L after adjustment for inflammation [63]. Iron deficiency data was used from a 2023 survey by Lebanon’s Ministry of Public Health [64]. Other clinical demand indicators used were prenatal, neonatal, and maternal deaths per 1000 newborns, pre-mature births and birth defects per 1000 live births [65], as well as total mandatory notifiable diseases (vaccine preventable, food and water borne, and other) per 100,000 people [66]. Capacity strain combined PHC centers per 100,000 people (Lebanese plus non-Lebanese people in 2019 [50]), calculated from published PHC counts [12], with the percentage of PHC centers meeting national staffing requirements surveyed from December 2017 to January 2018 [12]. Socioeconomic vulnerability was represented by displacement share (percent of governorate population that are non-Lebanese) [50] and household food insecurity, including mild, moderate, and severe categories [64] (Table A2).

### 2.2. Agent-Based Approach Using Biomimicry

In order to operationalize Vision 2030’s priorities, a qualitative Agent-Based Approach (ABA) was used for this preliminary policy analysis to improve Lebanon’s healthcare system and to suggest recommendations for system improvement [24]. Initially using ABA can lead to more quantitative analyses such as Agent-Based Modeling [24,67]. We decided to use ABA to help specify future ABM for improving Lebanese healthcare. Our ABA approach used biomimicry inspiration from the common grapevine (*Vitis vinifera*) to identify such potential areas for improvement. *Vitis vinifera* was selected over the common African baobab tree (*Adansonia digitate*) due to its adaptability and resilience under resource limitations (e.g., water; stretched PHC budgets needed for both locals and refugees). *Adansonia digitate* was more insightful to resource storage rather than resilience. How to improve resilience in healthcare as a Complex Adaptive System has recently been identified as a major research gap [68], which this current research hopes to fill. Using biomimicry for “societal transformation” (e.g., PHC reform) versus just for “innovation” by better engineering a particular attribute (e.g., burr inspiring Velcro) [46], we selected grapevine due to the complex, systemic processes it uses to conserve moisture in arid environments [69,70,71].

In *Vitis vinifera*, roots function as primary, distributed sensors of soil water deficit, detecting declines in soil water potential and contributing to systemic drought responses. As soil moisture declines, roots increase biosynthesis of abscisic acid, which is transported to the shoots and contributes to stomatal closure and reduced water loss. This responsiveness is underpinned by thousands of drought-responsive genes coordinating hormonal signaling, water transport through aquaporins, and structural remodeling of root tissues. This molecular plasticity enables roots to act as both sensors and messengers, translating localized water deficits into systemic signals that preserve hydraulic safety and coordinate whole-plant adaptation [69]. In order to be resilient to drought, grapevine root systems position new lateral roots closer (~3 cm versus 7 to 20 cm) to the main root axis, tightening connectivity to the strongest transport conduits. Thus new absorptive units remain closely coupled to major xylem pathways that sustain system-wide flow under stress. This proximity, redundancy, and direct connectivity prevents collapse [70]. During recovery, radial transport is restored near the root tip where laterals couple to the main axis, re-establishing functional connectivity at the most strategically important connection zone [71].

Lebanon’s PHC system was analyzed using the conceptual heuristic developed by Stafford Beer, which holds that “the purpose of a system is what it does, not what it is intended to do” (POSIWID) [24]. Rather than treating Lebanon’s PHC failures as implementation shortfalls against an idealized design, POSIWID analysis reframes them as emergent properties of a system that is, in effect, producing outcomes that reflect the actual interaction rules its agents follow (Current-Is-POSIWID). What Lebanon’s PHC system had been actually designed to do is then defined (Should-Be-POSIWID). The root difference is that between the “Current-Is” and “Should-Be” POSIWID.

The qualitative analysis in this study was structured in alignment with ABA’s two-phase framework. In the first phase, understanding the system, we synthesized the published literature, national health data, and publicly available institutional assessments to develop a working model of Lebanon’s PHC system. The biomimicry framework, specifically the drought-resilient root architecture of *Vitis vinifera*, served as the analytical lens through which this system’s understanding was developed. We identified three critical biomimicry functions [72] relevant to the common grapevine in arid conditions and used these characteristics to inspire improvements in Lebanon’s resource-scarce PHC system using this ABA methodology. Biological strategies were used to surface structural deficiencies that purely institutional or epidemiological analyses might otherwise treat as isolated technical failures.

In the second phase of the ABA, making recommendations, these structural deficiencies were translated into policy mechanisms by identifying the agents and simple interaction rules whose change would shift the system toward the goals articulated in Vision 2030. The analysis distinguished system-level agents, those who set the interaction rules governing Lebanon’s PHC system from operation-level agents, and those who follow those rules during day-to-day functioning. System-level agents include the MoPH, which sets governance frameworks, financing mechanisms, and accreditation standards; international agencies that conditionally finance a substantial share of PHC operations; and political actors who mediate access to healthcare resources through patronage networks. Specified operation-level agents included PHC centers, which serve as the frontline entry point to care as well as referral hospitals and secondary clinics, which receive escalated cases and are positioned to provide clinical oversight; NGOs, which operate the majority of PHC centers under MoPH contracts; healthcare providers; and patient populations, which exhibit bypass behavior reflecting the rules and incentives the system currently presents to them [24]. Root differences were produced by the interaction of these agents under their current rules, while policy recommendations target specific rule changes that can shift the system toward its desired outcome.

### 2.3. Complex Adaptive Systems Principles

Health systems have been characterized as Complex Adaptive Systems (CASs) in the health policy literature, where the framing explains why interventions that appear sound in design produce unintended or self-reversing effects in implementation [73,74,75]. CAS theory descriptively characterizes properties that systems exhibit but supplies no criteria for judging whether a proposed policy mechanism can operate within one [73,76]. Therefore, we defined six CAS principles to appraise if our policy recommendations for improving PHC in Lebanon satisfied these principles (Table A3). Five principles were derived by re-stating a descriptive CAS property as a prescriptive design requirement, a translation step with precedent in health policy analyses that use CAS properties to appraise system-strengthening and scale-up strategies [74,75]. The sixth principle is contextual and was added to ensure relevance to the national Vision 2030 strategy that the framework is intended to operationalize [23].

The following are the six key Complex Adaptive Systems principles structuring policy recommendations. Principle 1 is agent-level operability, specifying a change in what at least one agent does and not just a change in aggregate system output. Principle 2 is rule-based triggering, which must be activated by a predefined condition or threshold rather than by discretionary judgement alone. Principle 3 is feedback loop closure of an adaptive feedback loop between frontline signals and system-level responses. Principle 4 is non-reinforcement of discretionary allocation, which must not maintain, reinforce, or create new opportunities for politically or donor-driven resource allocation outside of needs-based criteria. Principle 5 is scalability without proportional resource increases implemented in a resource-constrained environment with no proportional expansion of budget or workforce to produce system-level effects. Finally, Principle 6 is alignment with at least one of Vision 2030’s five core strategic directions, namely governance, universal health coverage, service delivery, health security, and/or health promotion.

## 3. Results

**Figure 1:**
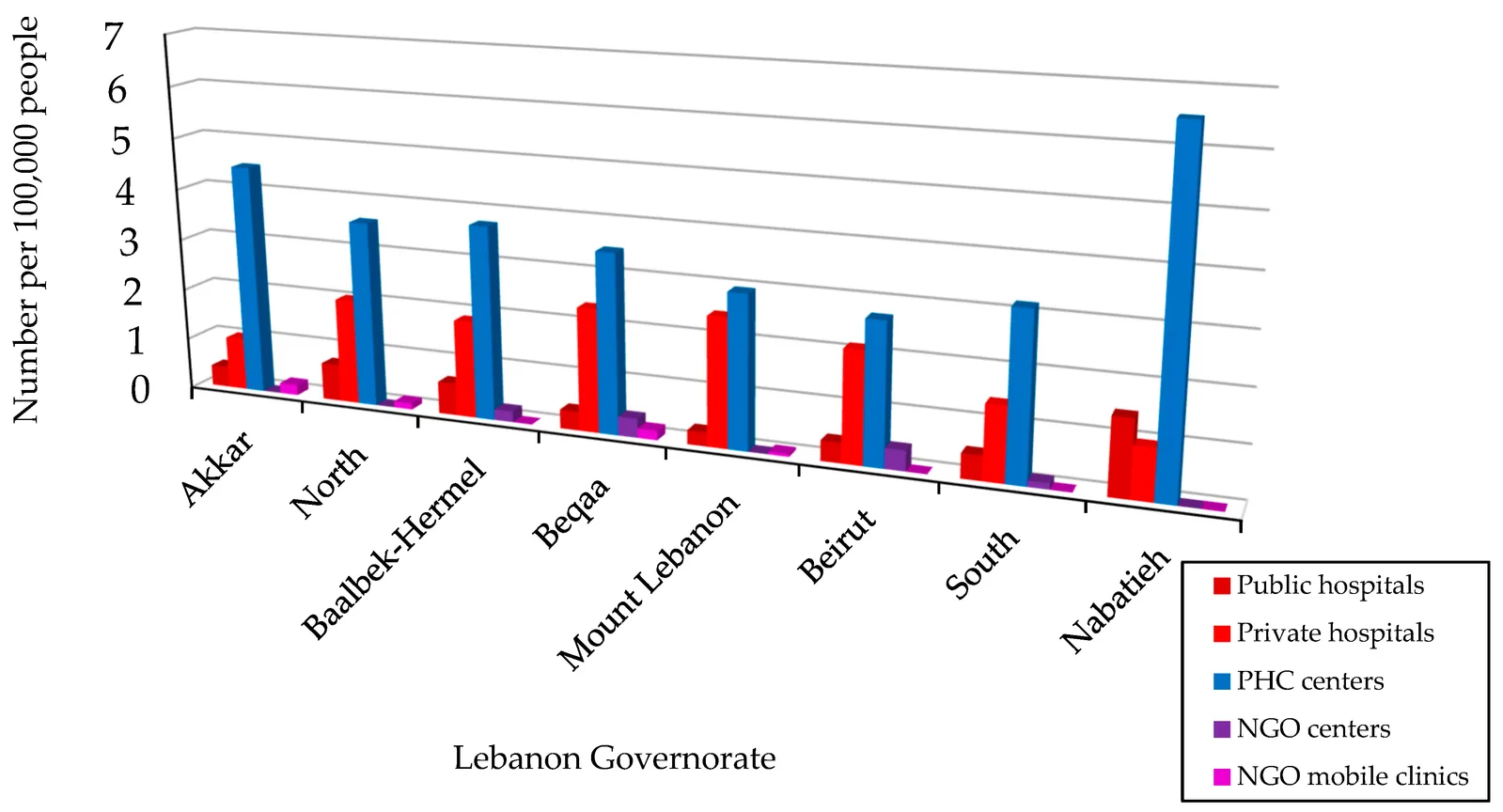
Hospital and primary healthcare (PHC) centers in 2017 and non-governmental centers and clinics in 2025 per 100,000 people (Lebanese plus non-Lebanese) in 2019.

As shown in Figure 1, peripheral governorates in Lebanon in the northern, eastern, and southern areas of the country outside of Beirut and Mount Lebanon have more PHC centers per 100,000 people, particularly Nabatieh (Table A1). Akkar governorate has a lower number of hospitals per 100,000 people compared to other regions (Figure 1). NGO centers are concentrated in Beqaa, Beirut, Baalbek-Hermel, and South governorates, while NGO mobile clinics are found in Beqaa, Akkar, North, and Mount Lebanon governorates (Figure 1). Focusing biomimicry inspired improvements to enhance the efficiency of Lebanon’s PHC system in Lebanon’s peripheral, underserved regions could address imbalances in healthcare.

**Figure A2:**
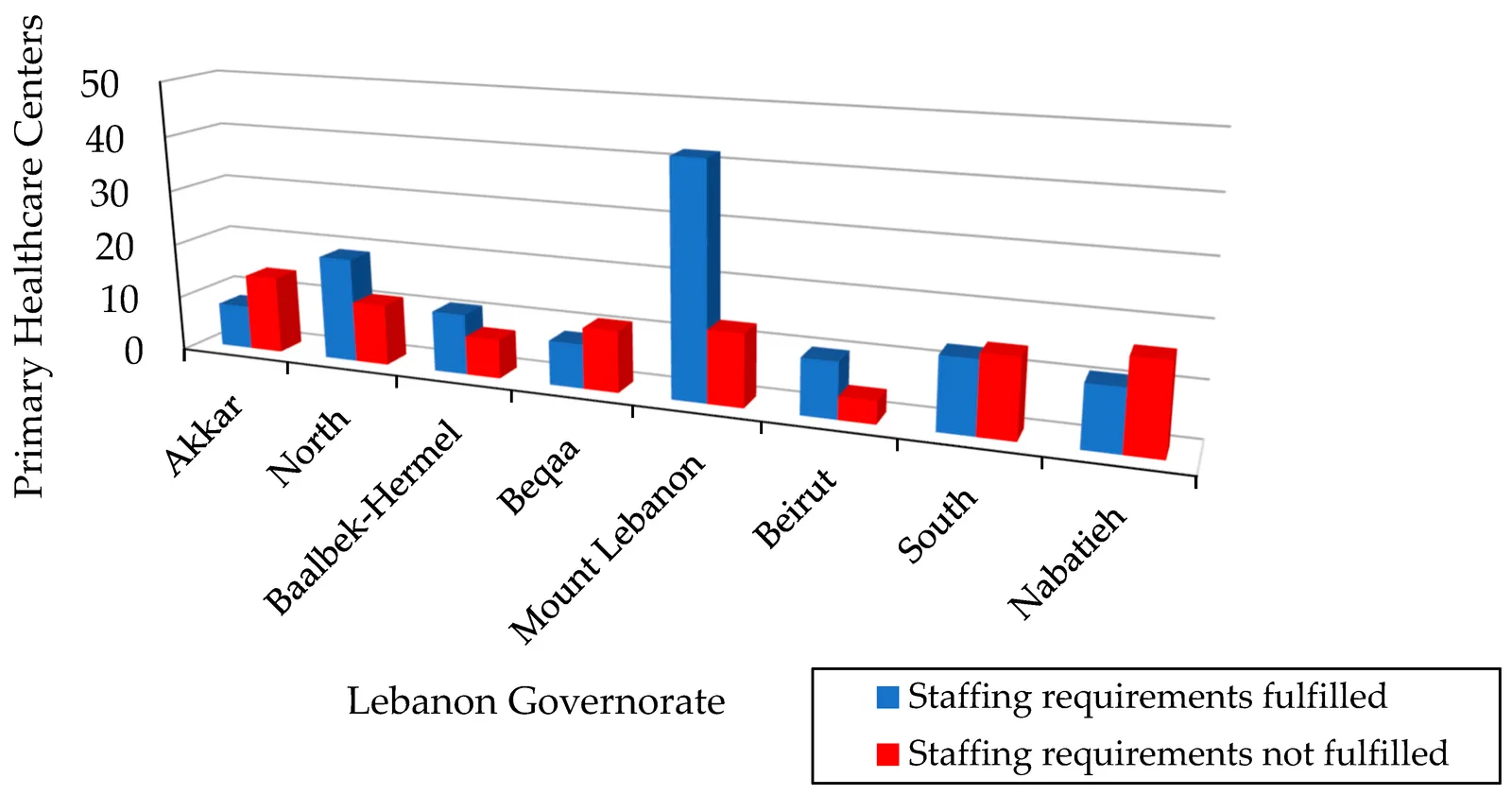
Primary healthcare centers with staffing requirements fulfilled and not fulfilled in 2017 [12].

**Figure A3:**
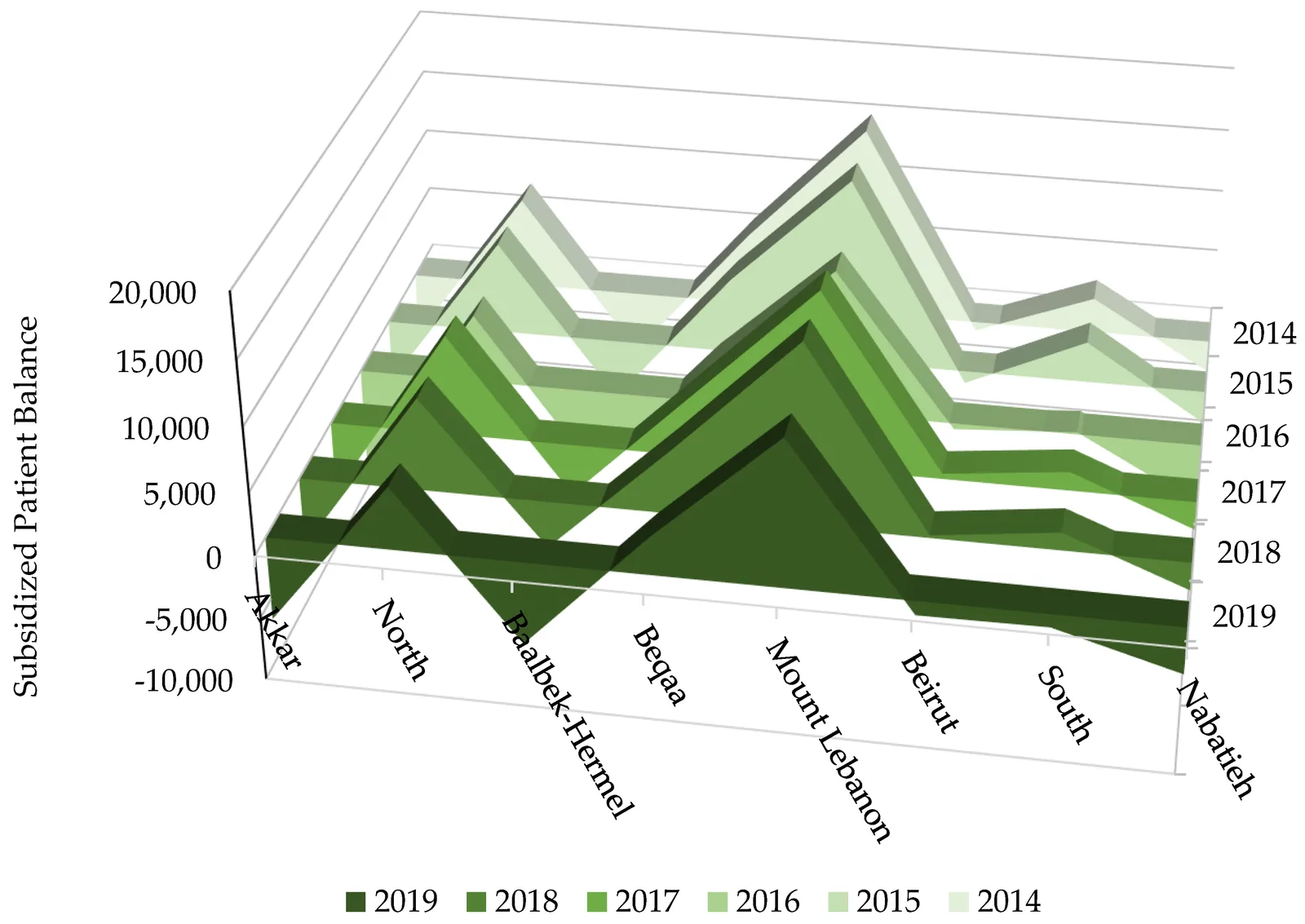
Subsidized patient balance calculated as subsidized PHC patients treated in hospitals in each municipality minus patients who are residents of each municipality from 2014 to 2019 [50].

While Lebanese governorates in the north, east, and south have a higher PHC density than Beirut and Mount Lebanon (Figure 1), services are not matched to patient demand. Governorates in these peripheral regions of Lebanon do not have adequate staffing. Staffing shortfalls in the Akkar, Beqaa, South, and Nabatieh governorates result in more PHC centers that do not have staffing fulfilled than do (Figure A2). When the number of subsidized patients living in each governorate exceeds the number of subsidized patients in hospitals in each governorate, then there is a negative subsidized patient balance. This suggests patients need to seek out health services elsewhere (e.g., PHC, NGOs, out-of-region hospitals). Governorates with a negative subsidized patient balance include Akkar, Baalbek-Hermel, Beirut, South, and Nabatieh (Figure A3).

### 3.1. Primary Healthcare Need Score

Our Need Scores are just preliminary calculations and do not reflect the relative ranking of the actual healthcare need of governorates in Lebanon. Preliminary primary healthcare Need Scores were calculated across Lebanon’s eight governorates of Akkar, Baalbek-Hermel, Beirut, Beqaa, Mount Lebanon, Nabatieh, North, and South (Table 1). This Need Score was the highest for Beqaa (69.2) followed closely by Akkar (66.4). Need Scores ranged from 29.1 to 59.5 for other governorates with Nabatieh having the lowest score (29.1). Beqaa’s Need Score was high due to high levels of disease (Table A1), particularly measles and viral hepatitis A, as well as having Lebanon’s greatest non-Lebanese population at 43% (Table A2). Akkar’s Need Score was elevated due to the highest levels of food insecurity (93.5%) and the lowest PHC staffing percentage (36.4%) of all governorates despite having the second highest PHC density (Figure 1) at 4.47 per 100,000 total Lebanese and non-Lebanese people (Table A2). Nabatieh has the highest PHC density at 6.53 per 100,000 people (Figure 1; Table A1).

Need Score output can be sensitive not only to indicators selected for all three domains but also to population and assigned domain and sub-domain weights. Need Scores varied more by *between-domain* weights compared to *within-capacity* weights, which only marginally changed relative Need Scores and only for lower –ranked governorates. The Need Score was calculated based on published Lebanese data across the eight governorates to establish whether it computes. Directional normalization, within-domain aggregation, and cross-domain aggregation behaved as designed, and every value is traceable from the published observation to composite output (Supplementary Materials—File S2). Calibrated weights, externally defined safe and crisis thresholds, and proper validation are prerequisites for using the Need Score as a policy trigger.

### 3.2. Agent-Based Approach Using Biomimicry

Table 2 applies the ABA’s POSIWID heuristic across the three structural domains (sensing and response, capacity distribution, referral integration) examined in this study, identifying the “Current-Is” purpose as revealed by its behavior, the desired “Should-Be” purpose based on Vision 2030’s goals and characteristics, and the root difference between the two purposes. Each root difference is examined in a section drawing on biomimicry principles derived from *Vitis vinifera* to translate the gap between current and desired system behaviors into specific design mechanisms. These improvements are summarized in Table 3.

#### 3.2.1. Process Information by Sensing Signals or Environmental Cues

Unlike the drought detection and response mechanisms of grapevine (*Vitis vinifera*) [69], Lebanon’s PHC system lacks institutionalized “sensing” mechanisms necessary for efficient, cost-effective, evidence-based care [8]. Key deficiencies include the absence of a unified healthcare information system across facilities, the lack of a national health registry, missing PHC-specific monitoring metrics, and non-interoperable data platforms that generate weak feedback loops, hindering real-time, data-informed decision-making [77,78]. This absence of adaptive feedback extends even to routine clinical practices, with 76.2% of PHC providers in Lebanon not implementing standardized screening, brief interventions, and referral for treatment for certain diseases, eliminating opportunities for early intervention and feedback-driven adjustment [79]. In the absence of institutionalized sensing mechanisms, resource allocation becomes fragmented across public, private, and non-governmental actors, leaving the public sector under-resourced and unable to coordinate evidence-based service delivery since there is no standardized, coordinated assessment of population need.

Once safe and crisis thresholds have been established and validated, a Need Score crossing the crisis threshold would trigger intensified outreach, mobile clinic services, rapid re-supply, and prevention programs. Intermediate values would activate scheduled outreach, while values in the safe range would sustain routine monitoring. Similar to the grapevine roots, Lebanon’s PHC centers should function as frontline active-sensing nodes, systemically measuring and reporting standardized signals that reflect population demand and service capacity. This includes the routine capture of (1) high-yield, feasible clinical screening indicators compatible with PHC workflows (e.g., treatment coverage gaps, health metrics), (2) service-capacity strain (e.g., PHC center per 100,000 people; percent staffing), and (3) population socioeconomic vulnerability (e.g., food insecurity). Each Need Score and its sub-scores normalized between safe and crisis thresholds can trigger such focused interventions.

#### 3.2.2. Modify by Adapting and Optimizing

Grapevines exhibit a highly plastic root architecture that is selectively distributed to optimize access to water across heterogeneous soil environments. Rather than expanding uniformly, *Vitis vinifera* allocates root biomass preferentially toward soil layers and zones where water availability is most reliable, enabling efficient resource capture under conditions of scarcity. Root systems extend vertically beyond 2 m while maintaining lateral spread, allowing vines to probe multiple hydrological niches and adjust absorptive presence according to local resource gradients [27,28]. Under drought conditions, grapevines actively reconfigure this architecture by reallocating growth toward deeper and more stable moisture zones, increasing root-to-shoot ratios and redistributing absorptive capacity away from depleted surface layers [26,32]. This depth-weighted distribution ensures that root presence is densest where water availability is highest or more persistent. Through this adaptive distribution strategy, grapevines maximize functional use of scarce resources while minimizing redundant investment in low-yield zones, achieving resilience through targeted expansion rather than uniform proliferation [30,69,80].

Just as grapevine root architecture is dynamically redistributed in response to sensed water availability, Lebanon’s PHC infrastructure must be reorganized using the spatial signals generated by Need Scores established in Section 3.1. Once PHC stress signals are translated into standardized Need Scores across districts, these signals should be used to guide how healthcare capacity is distributed geographically, ordering PHC centers along a gradient of measured need. Districts with the greatest epidemiological burden and access constraints with higher composite Need Scores would be prioritized for increased PHC presence and/or efficiency, staffing, and referral capacity. This would improve equity, resilience, and efficiency under persistent resource scarcity. Lower-need areas would shift toward maintenance rather than expansion. In addition to guiding expansion, Need Scores can inform what PHC services are deployed with care packages tailored to detected burdens. For example, where uncontrolled diabetes, vision loss, and vaccination gaps are clustered, PHC could integrate chronic-disease clinics and protect medicine supply chains. In this way, PHC expansion becomes targeted functional coverage, matching service intensity and service type to the local epidemiological gradient, rather than uniform proliferation.

#### 3.2.3. Get, Store, and Distribute Resources by Protective Positioning of Linkages

Lebanon’s PHC system lacks the structural positioning to serve as an integrated entry point to higher levels of care. PHC centers frequently operate with weak one-way linkages to referral hospitals, with no standardized, reliable, two-way referral pathways, feedback, nor continuity of care guiding patients between primary and secondary care [81], limiting quality improvement and clinical coordination [14,15]. Lack of electronic referral limits continuity and case management, with referral and counter-referral events going unrecorded, so that patients drift between facilities unchecked [12,14,82]. Additionally, shortages of family physicians (absent in 86.3% of PHC) and nurses (absent in 22%) undermine PHC’s ability to serve as a reliable referral anchor, contributing to widespread bypass behavior where patients seek direct access to more resource-intensive specialists or hospitals [5,12,13,16,83]. This is economically and clinically inefficient since a well-functioning PHC system resolves the health needs of 80% to 90% of the population and reserves secondary and tertiary care for more complex, higher intensity cases [84] by managing chronic and preventable cases [85].

Once Need Scores are defined and standardized across districts (Section 3.1), PHC expansion should be based on measured need and remain tightly coupled to at least one referral hospital or secondary clinic with relevant specialization. PHC should not be loosely connected, but rather anchored within this network through catchment-based pairing and standardized two-way referral loops that ensure escalation and back-referral, continuity, and feedback. Referral hospitals and clinics need to supervise, support, clinically oversee, provide referral feedback, and align protocols with their assigned PHC centers. Such connectivity can optimize scarce resources within defined geographic catchments with robust electronic records and communication. Lack of geographic proximity and electronic connectivity can result in patients bypassing PHC, overloaded hospitals, and referral delays, causing avoidable disease and death [86,87]. In this way, Lebanon’s PHC can better reflect the adaptive responses of grapevine root systems [70,71].

## 4. Discussion

### 4.1. International Precedents for Operationalizing Biomimicry-Derived Design Principles

We situate the biomimicry-derived PHC restructuring framework (Section 3.1 and Section 3.2) within international experience, using global precedents to demonstrate feasibility and operationalization in real systems (Table 4). Specifically, we highlight examples in which PHC has been strengthened through (1) standardized need sensing and national performance signals, (2) needs-based redistribution of capacity and services, and (3) protected referral architectures built around hub–spoke catchments, supervision, and two-way information flow. Together, these precedents illustrate how proposed Need Scores for Lebanon, adaptive redistribution logic, and core-anchored referral linkages can be practically implemented as policy rather than remaining as conceptual design goals.

#### 4.1.1. Process Information by Sensing Signals or Environmental Cues

Brazil implements Estratégia e-SUS Atenção Básica to capture routine PHC and Family Health Team signals, which are fed into a national monitoring system of local coverage and performance gaps to improve governance, financing, resource allocation, and evidence-based decision making [88,89]. Thailand uses standardized PHC indicators as both measurement tools and actionable signals triggering targeted service delivery [52]. Thailand’s National Health Security Office and Social Security Office Quality and Outcomes Frameworks use priority coverage measures (e.g., diabetes and hypertension monitoring and cervical cancer screening), enabling detection of missed screening and care gaps that prompt targeted outreach, adjusted delivery pathways, or incentive-linked reallocations [52]. Sweden’s Care Need Index was developed as a deprivation-weighted measure of expected primary-care workload and designed to guide the allocation of primary care resources across areas with varying levels of socioeconomic need [53]. This standardized scoring index has been incorporated into governance and financing, informing resource allocation and monitoring, and becoming a durable policy tool [54,55]. These international precedents can guide implementation of the preliminary Need Score for Lebanon that we have developed.

#### 4.1.2. Modify by Adapting and Optimizing

England uses need-weighted allocation for community and primary care staffing, planning, expansion, and resources, which operationalizes evidence-based equity as a routine decision rule [56,57]. Ghana situated community health resources within remote and rural populations and paired them with outreach, referral facilitation, and preventive services, which reduced geographic barriers to care and improved healthcare services, engagement, utilization, and outcomes [90,91]. By reallocating community health workers based on travel-time accessibility and modeled disease burden using geospatial modeling, Mali maximized geographic coverage and service reach without a proportional increase in workforce size, improving efficiency and equity simultaneously [92]. These international cases demonstrate that need-based redistribution can be institutionalized through allocation rules, targeted placement, and geospatial optimization, such as for the proposed PHC expansion and improved efficiency and service delivery in Lebanon.

#### 4.1.3. Get, Store, and Distribute Resources by Protective Positioning of Linkages

Kenya uses a modified hub-and-spoke model characterized by PHC facilities (spokes) managed and supported within the catchment area by a PHC referral facility (hub) utilizing e-health, planned accessibility, and both upward and downward referrals with feedback to the referral site, as well as network tracking [93]. In Cuba, district-level hubs integrate diagnostics, specialist services, surveillance, and supervision for multiple peripheral primary care units where each polyclinic functions as an anchor for a defined catchment for referral reception and ongoing clinical oversight, which shortens escalation pathways and preserves continuity across levels of care, buffering system stress [94]. In Tanzania’s Kilolo district, weak functional anchoring between primary health dispensaries and secondary facilities contributed to by-pass behavior and disproportionate strain on higher-level hospitals, while in the Msalala district, closer and more reliable anchorage to secondary hubs preserved referral flow and reduced upstream overload [95]. Key attributes of these international cases can be applied to Lebanon’s PHC.

### 4.2. Public Policy Recommendations

This study advocates for an operational framework for advancing Lebanon’s Vision 2030’s goals by translating biomimicry principles derived from the drought-adapted root system of *Vitis vinifera* into healthcare assessment and policy mechanisms for PHC reform. Rather than treating biomimicry as metaphor, we use it as design logic to structure monitoring and governance under chronic scarcity under the three structural domains in Table 2. All recommendations were evaluated against six Complex Adaptive Systems-grounded principles noting where compliance is full or partial and flagging areas for future refinement as the framework moves toward implementation (Table A4).

#### 4.2.1. Sensing and Response

Lebanon’s PHC system lacks institutionalized sensing mechanisms capable of translating frontline stress into coordinated system-level adaptations. Resource allocation is instead shaped by donor priorities, political discretion, and inherited infrastructure rather than measured need (e.g., Need Scores). By contrast, grapevine root systems operate as distributed sensors that detect localized water stress and rapidly transmit signals that trigger systemic adjustment before hydraulic failure occurs. Translating this principle, PHC facilities must function as frontline detectors whose signals inform governance decisions in real time. Need Scores operationalize this logic by converting epidemiological burden and service-capacity strain into standardized, comparable stress indexes.

Policy Recommendation 1: Lebanon’s MoPH needs to refine and adopt national PHC Need Scores by improving and using our Need Score calculator (Supplementary Materials—File S2) as a model prototype. Need Scores need to be reported at regular intervals by all PHC centers within the national network. This score should integrate a limited set of high-yield indicators from three domains: (1) clinical demand (e.g., health challenges), (2) service-capacity strain (e.g., PHC capacity, staffing shortages), and (3) socioeconomic vulnerability (e.g., displaced populations, food insecurity). Need Scores require normalization against predefined safe and crisis thresholds, triggering with response packages based on score ranges. Embedding Need Scores within Vision 2030’s monitoring framework would shift governance from discretionary allocation to rule-based, adaptive management.

This recommendation satisfies all six Complex Adaptive Systems principles (Table A4). Need Scores operationalize agent-level rule changes at the PHC-center level (Principle 1), activate responses through predefined thresholds (Principle 2), close the feedback loop between frontline stress signals and governance decisions (Principle 3), replace discretionary allocation with standardized triggers (Principle 4), and leverage existing PHC reporting infrastructure rather than requiring new institutional capacity (Principle 5). It directly advances Vision 2030’s governance and service delivery directions (Principle 6).

#### 4.2.2. Capacity Distribution

Resilient systems must redistribute capacity toward the zones of highest functional need. Lebanon’s PHC distribution exhibits the opposite pattern. The central parts of Lebanon (e.g., Beirut and Mount Lebanon) tend to have hospitals, PHC centers, and NGOs that are denser (Figure 1) with higher staffing percentages (Table A1) compared to other regions (Figure 1). Therefore, the MoPH should use district-level Need Scores to further guide where and how PHC capacity is expanded and/or better staffed to better serve patients.

Policy Recommendation (P.R.) 2: The MoPH needs to translate Need Scores into spatial expansion plans guiding PHC site location, intensity of operation, staffing, and services offered, addressing regions (e.g., northern, eastern, and southern Lebanon) with high demand, inadequate healthcare services, and resource-challenged populations (Figure 1). P.R. 2.1: In districts with high Need Scores, improved healthcare services need to be prioritized. P.R. 2.2: In districts where PHC facilities already exist but exhibit persistently high Need Scores, PHC sites need to be re-scaled and reconfigured (e.g., altering/redistributing staffing, operating hours, diagnostic and pharmacy capacity, and service packages). Lower-need districts should shift towards maintenance, consolidation, and efficiency improvements rather than new construction or expansion of services.

This recommendation satisfies five of the six Complex Adaptive Systems principles. It specifies rule changes for the MoPH as the system-level agent governing spatial planning decisions (Principle 1), tying redistribution decisions to Need Scores rather than discretionary judgment (Principles 2 and 4). It contributes to feedback loop closure by ensuring sensing signals translate into physical capacity changes (Principle 3) and aligns directly with Vision 2030’s universal health coverage and service delivery direction (Principle 6). There is partial compliance with Principle 5 since initial siting or construction of new PHC access points in high-need districts requiring capital investment that may be resource-intensive in the short term (Table A4). However, reallocation mechanisms limit proportional net increases in budget and/or workforce, ensuring compliance with Principle 5 over time.

#### 4.2.3. Catchment and Referral Integration

Sensing and redistribution alone are insufficient if PHC facilities are loosely connected. Lebanon’s PHC system lacks supervised coupling to secondary care and reliable feedback through referral and counter-referral loops. Referral pathways between PHC centers and hospitals are inconsistent and weakly tracked, undermining PHC as a reliable entry point. When escalation routes are uncertain and counter-referral is sporadic, care continuity breaks at transition points, hospital demand rises, and PHC teams lose access to clinical information needed to support follow-up, learning, and quality improvement.

Policy Recommendation 3: Lebanon’s MoPH should formalize a primary care network architecture where every PHC center is explicitly paired with a designated referral hospital or secondary clinic as a supervisory hub. These pairings should define a catchment, involve minimum hub obligations including clinical supervision, scheduled specialist outreach, guaranteed escalation pathways, and mandatory counter-referral of care.

This recommendation satisfies five of six CAS principles. Formalizing catchment base pairing changes the interaction rules between PHC centers and referral hospitals as agent pairs (Principle 1) and defining minimum hub obligations creates rule-based rather than discretionary supervisory relationships (Principles 2 and 4). The supervised hub–spoke architecture structurally anchors feedback between operational levels (Principle 3), aligning with Vision 2030’s service delivery and governance directions (Principle 6). Principle 5 is potentially resisted and partially complied with since establishing supervisory relationships and specialist outreach requires reallocating hospital capacity (Table A4). This is most feasible where existing hospital–PHC geographic proximity already exists.

Policy Recommendation 4: The MoPH needs to implement a national electronic referral and counter-referral system that tracks referral initiation, acceptance, service delivery, and return of information to PHC centers. Referral loops should be considered complete only when counter-referral is documented. Financing and contracting mechanisms should link performance incentives to referral-loop completion, supervision activities, and continuity-of-care indicators. Referral data must feed back into Need Scores, closing the adaptive feedback loop between sensing, response, and outcomes.

This recommendation satisfies all six principles (Table A4). An electronic referral and counter-referral system directly changes the interaction rules governing information flow between PHC centers and referral hospitals (Principle 1). Linking performance incentives to referral-loop completion creates rule-based accountability triggers (Principle 2). Feeding referral data back into Need Scores closes the adaptive feedback loop across all three structural domains (Principle 3); removes informal referral practices that currently enable discretionary patient routing (Principle 4); and leverages the digital infrastructure investments already anticipated under Vision 2030’s digital health agenda, rather than requiring new standalone systems (Principles 5 and 6).

### 4.3. Practical Implications and Limitations

Effective reform depends on strengthening the “4 Cs” of primary care: first-contact access, continuity, comprehensiveness, and coordination, supported by governance, financing, workforce, and digital infrastructure [96,97,98]. The 2018 Declaration of Astana reaffirmed these priorities, calling for PHC systems that are people-centered, equity-driven, and digitally connected to guarantee two-way referrals between community and hospital levels [99]. Lebanon’s PHC system has been incredibly resourceful in accommodating Syrian refugees [100] while maintaining extremely high patient satisfaction [101]. NGO community-based care has also been supportive [102]. However, PHC expansion and/or efficiency enhancements without embedded data-driven adaptation limits gains, requiring resilient PHC core operating principles such as measurement, feedback, and course correction [103,104]. Improving upon our preliminary Need Score and following our proposed policy framework can enhance Lebanon’s historical data managements systems [105] and efficiency analyses [7]. This can complement the use of financial indicators by hospitals [106] as well as using advanced digital technologies to streamline healthcare efficiency [107] to improve PHC catchment and referral integration. Enhancing PHC centers can accompany universal healthcare, reducing excessive patient readmissions, and implementing telemedicine in Lebanon [108].

Although contextually developed for Lebanon’s Vision 2030, our framework may be relevant to other fragile and crisis-prone settings. Our research advances an Agent-Based Approach using biomimicry as a framework for improving health-systems architecture and governance. This pushes biomimicry upstream into public-sector research design [109], suggesting that biological resilience principles can inform how Complex Adaptive Systems (e.g., healthcare systems) learn, self-correct, and preserve continuity over time. We used general biological strategies that increase *Vitis vinifera*’s resilience to water scarcity to inspire policy frameworks to improve PHC in Lebanon. Such application of biomimicry does not necessarily mean there is a literal relationship between grapevine’s biology and Lebanese PHC, rather that biomimicry can be used to think outside of the box both literally and figuratively to develop more regenerative social systems.

Our current study proposes a conceptual and policy-design framework using a preliminary version of a Need Score for PHC in Lebanon, not an implementation trial nor a validated instrument. Future research needs to quantitatively validate our Need Score (e.g., indicator selection, weighting, thresholds, and sensitivity to missing data) and define the operational details of the rollout of healthcare enhancements (cost, workforce requirements, procurement pathways, and enforcement mechanisms). Empirical calibration will require prospective piloting in selected districts, assessment of data quality and feasibility, and iterative refinement based on observed performance. Lebanon’s current compromised health sector may limit the pace and uniformity of reform. Also, patient-level outcomes, cost-effectiveness, and equity impacts need assessment as reforms are operationalized.

Our Need Score example has limitations. Observed-range normalization is sample-dependent, while all weights are transparent defaults rather than calibrated parameters. The resulting values must therefore be validated before becoming reliable estimates of regional need and resource allocation. Operationalization will require validation of indicators and crisis thresholds (Supplementary Materials—File S1: Table S2), sensitivity analyses for missing data and temporal drift, and calibration of within-domain and between-domain weights against observed workload, unmet-need, or preventable-outcome criteria, following the logic of Sweden’s Care Need Index [53]. Also, population figures for both Lebanese and non-Lebanese individuals are from 2019 and may not reflect more recent refugees from current regional conflicts and forced displacements.

## 5. Conclusions

Lebanon’s primary healthcare (PHC) system exhibits chronic scarcity, political fragmentation, and recurrent shock, rendering conventional, expansion-focused reform strategies insufficient. Resilience under such constraints depends less on scale than on architecture: how systems sense stress, redistribute capacity, protect core functions, and adapt over time. Drawing on the drought-resilient root system of *Vitis vinifera*, we advance a biomimicry-inspired policy framework that uses biological resilience principles to guide operational mechanisms for PHC governance, spatial planning, and referral integration. Distributed sensing needs to be operationalized through improving the preliminary, standardized PHC Need Score that we have developed in order to convert frontline epidemiological and service-capacity signals into rule-based policy responses. Adaptive redistribution replaces static, historically inherited infrastructure patterns with the need-responsive deployment of PHC presence and service packages. Structural anchoring repositions PHC as a true entry point to care by coupling centers to supervised referral hubs through defined catchments and electronic referral and counter-referral loops. Together, these mechanisms enable PHC to function as an adaptive, learning system capable of preserving continuity of care while protecting higher-level resources.

This approach directly supports the objectives of Lebanon’s Vision 2030 by shifting governance from discretionary allocation toward transparent, evidence-informed decision-making. This can strengthen PHC’s role, and improve system resilience under fiscal and political constraints. This can be a prescriptive blueprint not just for Lebanon, but for any fragile and crisis-prone health system where PHC networks exist yet fail to function as coordinated, adaptive infrastructure. Future research can quantitatively model PHC using Agent-Based Modeling, further validate PHC Need Scores, assess if response packages measurably improve continuity and equity compared to discretionary allocation, and evaluate the impact of hub–spoke catchments with electronic referral and counter-referral trackers on patient outcomes and hospital loads. Such biomimicry-informed public policy designs can optimize Complex Adaptive Systems like healthcare that are faced with routine scarcity.

## Figures and Tables

**Table 1:** Normalized domain values and composite output of the worked computation, by governorate. Values demonstrate Need Score computation and are not a ranking of healthcare need.

Governorate	Clinical Demand (C)	Capacity Strain (S) ^a^	Socioeconomic Vulnerability (V)	Need Score (0 to 100)
Akkar	0.423	0.769	0.801	66.4
Baalbek-Hermel	0.680	0.557	0.511	58.3
Beirut	0.515	0.563	0.297	45.8
Beqaa	0.507	0.830	0.740	69.2
Mount Lebanon	0.557	0.469	0.107	37.7
Nabatieh	0.137	0.446	0.290	29.1
North	0.158	0.549	0.454	38.7
South	0.586	0.792	0.408	59.5

^a^ Equal weighting of density (50%) and staffing (50%) capacity strains.

**Table 2:** Purpose Of the System Is What It Does (POSIWID) differentiation and root difference analysis of Lebanon’s primary healthcare system.

Structural Domain	Current-Is-POSIWID (What the System Actually Does)	Root Difference	Should-Be-POSIWID (What the System Needs to Do)
Sensing and response	Lebanon’s primary healthcare (PHC) system is designed for operating without standardized need signals, producing resource allocation shaped by donor priorities, political discretion, and inherited infrastructure.	Absences of institutionalized sensing mechanisms and rule-based threshold responses.	Sense frontline stress signals through standardized indicators, aggregate them into comparable Need Scores, and activate predefined responses aligned with measured population need.
Capacity distribution	Lebanon’s PHC system is designed for concentrating healthcare capacity in urban and politically connected areas, producing persistent misalignment between service availability and epidemiological burden across districts.	Absence of need-responsive redistribution rules governing where and how PHC capacity is deployed.	Redistribute capacity such as staffing, services, and physical presence, dynamically toward districts with highest measured need.
Catchment and referral integration	Lebanon’s PHC system is designed for one-way, untracked patient movement toward hospitals and specialists, producing bypass of primary care, erosion of care continuity, and avoidable overload of secondary services.	Absences of structural anchoring between PHC centers and referral hubs through defined catchments and two-way information flow.	Anchor every PHC center to a designated referral hub through defined catchments, supervised escalation pathways, and electronic counter-referral loops that preserve continuity and protect secondary resources.

**Table 3:** Biomimicry functions of *Vitis vinifera* applied to Lebanese primary healthcare to address root differences.

Biomimicry Functions	Grapevine (*Vitis vinifera*)	Lebanese Primary Healthcare (PHC)
Process information by sensing signals or environmental cues	Root system sensing mechanisms allow grapevine to adapt to drought.	PHC Need Scores address absences of institutionalized sensing mechanisms and rule-based threshold responses.
Modify by adapting and optimizing	Selectively reallocates root growth toward better-hydrated soil zones optimizing resource capture under water stress.	Redistributes facilities, services, and resources toward districts with higher measured Need Scores using need-responsive redistribution rules governing where and how PHC capacity is deployed.
Get, store, or distribute resources by protective positioning of linkages	Positions lateral roots close to main root axis during drought, maintaining tight coupling to core xylem pathways.	PHC centers tightly anchored to referral hospitals within defined catchments and two-way referral systems so there is now structural anchoring between PHC centers and referral hubs through defined catchments and two-way information flow.

**Table 4:** Restructuring to improve primary healthcare in Lebanon using biomimicry characteristics and global examples on which to model such improvements.

Biomimicry Functions	Lebanese Primary Healthcare (PHC) Restructuring	Global Examples
Process information by sensing signals or environmental cues	PHC centers function as frontline sensing nodes that detect population health demand and service-capacity strain.	Estratégia e-SUS Atenção Básica Primary Care platform, Brazil [88,89]. National Health Security Office and Social Security Office Quality and Outcomes Framework, Thailand [52]. Care Need Index, Sweden [53,54,55].
Modify by adapting and optimizing	Dynamic redistribution of PHC presence, staffing, and services in proportion to measured demand–supply gaps.	Need-weighted allocation formulas guide primary care staffing and resources, England [56,57]. Community-based Health Planning and Services initiative, Ghana [90,91]. Geospatial matching of healthcare workers to underserved areas, Mali and Sierra Leone [92].
Get, store, or distribute resources by protective positioning of linkages	PHC anchorage with defined catchments and two-way referrals.	Formal hub-and-spoke PHC networks with geographic and electronic referral coupling in Kenya [93]. Polyclinic model linked family doctor offices to hospital-grade services, Cuba [94]. Closer anchorage to secondary hubs preserved flow and reduced burdens, Msalala versus Kilolo, Tanzania [95].

## Data Availability

The data presented in this study are available on request from the corresponding author due to privacy.

## Supplementary Materials

The following supporting information can be downloaded at: https://www.mdpi.com/article/10.3390/healthcare14172741/s1, Supplementary Materials—File S1: Table S1: Need Score equations and example specifications for Akkar governorate, Lebanon; Table S2: Need Score indicator examples with domain assignment and weighting, primary healthcare (PHC)-level collection mechanism, threshold, and threshold source for each indicator, Supplementary Materials—File S2: Need Score—Full Calculation Workbook.

## Appendix A

**Table A1:** Clinical demand indicators of (1) iron deficiency, (2) prenatal, neonatal, and maternal deaths, and (3) pre-mature births and birth defects for Lebanon’s eight governorates.

Governorate	Iron Deficiency (%) ^a^ [64]	Still Births, IUFD, Neonatal Deaths, and Maternal Deaths per 1000 Newborns ^b^ [65]	Pre-Mature Births and Birth Defects per 1000 Live Births ^b^ [65]	Total Diseases ^c^ per 100,000 Lebanese and non-Lebanese ^d^ [50,66]
Akkar	32.0	23.5	88.4	67.7
Baalbek-Hermel	23.6	27.2	111.3	158.3
Beirut	50.7	12.2	117.8	42.8
Beqaa	34.2	20.0	80.4	140.3
Mount Lebanon	41.2	23.6	107.1	51.4
Nabatieh	23.7	19.5	60.3	52.9
North	32.2	14.1	72.5	35.5
South	36.5	30.2	109.3	37.4
Average	36.1 ^e^	21.3 ^e^	93.4 ^e^	73.3 ^e^

^a^ From 2023 survey by Lebanon’s Ministry of Public Health (MoPH) [64]. ^b^ Lebanon MoPH’s vital data observatory statistics. ^c^ Lebanon MoPH’s surveillance data. ^d^ Ministry of Public Health’s E-Bulletin Statistics 2019 [50]. ^e^ Indicators share-weighted average weighted by population.

**Table A2:** Primary healthcare (PHC) centers and densities, Lebanese and non-Lebanese populations, and displacement share and food insecurity for Lebanon’s eight governorates.

Governorate	Primary Healthcare Centers ^a^ [12]	Staffing Fulfilled (%)	Lebanese Population ^b^ [50]	Non-Lebanese Population ^b^ [50]	PHC Centers per 100,000 Total People ^c^	Displacement Share ^b^ (%) [50]	Food Insecurity (%) ^d^ [64]
Akkar	22	36.4	338,827	153,044	4.47	31.1	93.5
Baalbek-Hermel	18	61.1	349,846	131,370	3.74	27.3	70.0
Beirut	14	71.4	421,624	94,317	2.71	18.3	63.5
Beqaa	19	42.1	312,452	235,496	3.47	43.0	66.5
Mount Lebanon	55	76.4	1,568,712	294,863	2.95	15.8	48.0
Nabatieh	27	40.7	359,356	54,339	6.53	13.1	71.7
North	30	63.3	664,393	171,263	3.59	20.5	75.9
South	27	48.2	531,408	318,648	3.18	37.5	41.6
Average	26.5	57.6 ^e^	568,327	181,668	3.53	25.8	61.1 ^e^
Lebanon Total	212	-	4,546,618	1,453,340	-	-	-

^a^ Hemadeh et al. (2020) survey of PHC from December 2017 to January 2018 [12]. ^b^ Ministry of Public Health’s E-Bulletin Statistics 2019 [50]. ^c^ PHC centers per 100,000 total Lebanese and non-Lebanese. ^d^ From 2023 survey by Lebanon’s Ministry of Public Health [64]. ^e^ Staffing share-weighted average weighted by PHC centers. Food insecurity share-weighted average weighted by population.

**Table A3:** Derivation and provenance of six appraisal criteria for Complex Adaptive Systems applied to Lebanon’s healthcare policy recommendations.

Complex Adaptive System (CAS) Appraisal Criterion ^a^	Derivation Where Descriptive Property Restated as a Design Requirement	Source(s)
1. Agent-level operability	System behavior arises from agents acting on local rules Mechanism must specify change in what one identified agent does Not just a change in aggregate output	[24,73,76]
2. Rule-based triggering	Simple local rules generate system-level order Action must be triggered by a predefined condition or threshold Triggering of action not by discretionary judgement	[55,56,76,91,92]
3. Feedback loop closure	Feedback is a defining property of adaptive systems Systems structurally linked by measuring, feedback, and course correction Loop must close between frontline signals and system-level response	[73,100,101]
4. Non-reinforcement of discretionary allocation	Path dependence restores established configuration unless disrupted In Lebanon, there is patronage- and donor-mediated allocation Must not maintain, reinforce, or create allocation that is not need-based	[8,9,11,74]
5. Scaling without proportional resource increase	Nonlinearity permits system-level effect without proportional input Precondition for reform under fiscal constraint Mechanism must produce effects without budget or workforce expansion	[73,74,75]
6. Alignment with Vision 2030	Not derived from a CAS property and criterion are contextual Must address at least one of the five Vision 2030 strategic directions: (1) Governance, (2) Universal Health Coverage, (3) Service Delivery, (4) Health Security, and/or (5) Health Promotion	[23]

^a^ Criteria 1 through 5 were constructed for this study by restating a descriptive property of Complex Adaptive Systems as a design requirement, while criterion 6 is contextual and is not derived from a systems property.

**Table A4:** Complex Adaptive Systems recommendations rubric and compliance summary across policy recommendations.

Complex Adaptive System Principle	Policy Recommendation 1: Need Scores	Policy Recommendation 2: Expansion Plans	Policy Recommendation 3: Network Architecture	Policy Recommendation 4: Electronic Referral
Agent-level operability	✓	✓	✓	✓
2. Rule-based triggering	✓	✓	✓	✓
3. Feedback loop closure	✓	✓	✓	✓
4. Non-reinforcement of discretionary allocation	✓	✓	✓	✓
5. Scalability without proportional resource increase	✓	◑	◑	✓
6. Alignment with Vision 2030 strategic directions	✓	✓	✓	✓
Overall compliance	Full	Partial	Partial	Full

✓ Full compliance—◑ Partial compliance.
