# Supplementary material for: Improving Primary Healthcare: Applying Grapevine (Vitis vinifera) Principles to Lebanon’s Vision 2030
Source: Healthcare (Basel). 2026 Aug 28;14(17):2741. doi: 10.3390/healthcare14172741 (PMC13565197; doi:10.3390/healthcare14172741)
Supplement: Supplementary file 1 [file healthcare-14-02741-s001.zip › Supplementary Materials - File S1 - Final3.pdf]

Research

Improving Primary Healthcare: Applying Grapevine (*Vitis vinifera*) Principles to Lebanon’s Vision 2030

Karim W. Barake [1,2], Aaron Kinyu Hoshide [1,3,4,*] , Salim M. Adib [5] and Kimberly Samaha [1]

[1] Born Global Foundation, 254 Commercial Street, Portland, ME, 04101, USA; ahoshide@bornglobalfoundation.org (A.K.H.); ksamaha@bornglobalfoundation.org (K.S.)
[2] Kilachand Honors College, Boston University, Boston, MA 02215, USA; barake@bu.edu
[3] Sensei Economic Solutions, LLC, Rome, GA 30165, USA; sensei@senseieconomics.com
[4] AgriSciences, Instituto de Ciências Agrárias e Ambientais, Universidade Federal de Mato Grosso, Sinop 78555-267, MT, Brazil
[5] Department of Epidemiology and Biostatistics, American University of Beirut, P.O. Box 11-0236, Beirut 1107 2020, Lebanon; sa193@aub.edu.lb
* Correspondence: ahoshide@bornglobalfoundation.org; Tel.: +1-207-659-4808

Supplementary Materials – File S1

Table S1. Need Score equations and example specifications for Akkar governorate, Lebanon.

Equation	Specification	Definitions	Akkar Governorate, Lebanon
Need Score (NS)	$NS_d = 100(w_C C_d + w_S S_d + w_V V_d)$	d = governorate<br>w = domain weight	$NS_{\text{Akkar}} = 100\left(\frac{0.423 + 0.769 + 0.801}{3}\right) = 66.4$
Sub-Scores			
Clinical Demand (C)	$C_d = \sum_i \alpha_i \tilde{n}(c_{i,d})$	C_d = Weighted clinical demand strain by governorate<br>ñ = min-max normalization<br>α_i = within domain weight for C	$CD_{\text{Akkar}} = 0.25(0.31) + 0.25(0.63) + 0.25(0.489) + 0.25(0.262) = 0.423$
		ID_d = Normalized iron deficiency (%) for children ages 6 to 59 months	$ID_{\text{Akkar}} = \frac{32.0 - 23.6}{50.7 - 23.6} = 0.310$
		$PNMD_d$ = Normalized prenatal, neonatal, and maternal deaths per 1,000 newborns	$PNMD_{\text{Akkar}} = \frac{23.5 - 12.2}{30.2 - 12.2} = 0.630$
		$PMBBD_d$ = Normalized pre-mature births and birth defects per 1,000 live births	$PMBBD_{\text{Akkar}} = \frac{88.4 - 60.3}{117.8 - 60.3} = 0.489$
		TD_d = Normalized total diseases per 100,000 Lebanese and non-Lebanese	$TD_{\text{Akkar}} = \frac{67.7 - 35.5}{158.3 - 35.5} = 0.262$
Service-Capacity Strain (S)	$S_d = \sum_j \beta_j \tilde{n}(s_{j,d})$	S_d = Weighted service-capacity strain by governorate<br>β_j = within domain weight for S	$S_{\text{Akkar}} = 0.50(0.5386) + 0.50(1.000) = 0.769$
		D_d = Normalized density strain	$D_{\text{Akkar}} = \frac{6.527 - 4.473}{6.527 - 2.713} = 0.5386$

Equation	Specification	Definitions	Akkar Governorate, Lebanon
		H_d = Normalized staffing strain	$H_{\text{Akkar}} = \frac{76.4 - 36.4}{76.4 - 36.4} = 1.000$
Socioeconomic Vulnerability (V)	$V_d = \sum_k \gamma_k \, \tilde{n}(v_{k,d})$	γ_k = within domain weight for V	$V_{\text{Akkar}} = 0.50(0.602) + 0.50(1.000) = 0.801$
		DS_d = Normalized displacement share (% of population non-Lebanese)	$DS_{\text{Akkar}} = \frac{31.1 - 13.1}{43.0 - 13.1} = 0.602$
		FI_d = Normalized food insecurity (% of households that are food insecure)	$FI_{\text{Akkar}} = \frac{93.5 - 41.6}{93.5 - 41.6} = 1.000$
Domain Weights	$w_C + w_S + w_V = 1$	w_C = domain weight for C<br>w_S = domain weight for S<br>w_V = domain weight for V	1/3 + 1/3 + 1/3 = 1
Within Domain Weights	$\sum_i \alpha_i = 1$	α_i = within domain weight for C	$\sum_i \alpha_i = 1/4 + 1/4 + 1/4 + 1/4 = 1$
	$\sum_j \beta_j = 1$	β_j = within domain weight for S	$\sum_j \beta_j = 1/2 + 1/2 = 1$
	$\sum_k \gamma_k = 1$	γ_k = within domain weight for V	$\sum_k \gamma_k = 1/2 + 1/2 = 1$
Indicator Common Scales			
Worse if Rise	$\tilde{n}(x) = clip\,(\frac{x - \tau_{safe}}{\tau_{crisis} - \tau_{safe}}, 0, 1)$	$\tilde{n}(x)$ = min-max normalization for observation x	τ_{safe} = 76.4<br>τ_{crsis} = 36.4
Worse if Fall	$\tilde{n}(x) = clip\,(\frac{\tau_{safe} - x}{\tau_{safe} - \tau_{crisis}}, 0, 1)$	τ_{safe} = Safe threshold<br>τ_{crsis} = Crisis threshold	$H_{\text{Akkar}} = \frac{76.4 - 36.4}{76.4 - 36.4} = 1.000$

Table S2. Need Score indicator examples with domain assignment and weighting, primary healthcare (PHC)-level collection mechanism, threshold, and threshold source for each indicator.

Domain (weight)	Indicator	Precise Metric	Collection Mechanism	Threshold (τ_j)	Direction
**D_1 — Clinical Demand (w_C)**	Iron Deficiency	Iron deficiency among children ages 6 to 59 months with plasma ferritin < 12 µg/liter (%)	2023 survey by Lebanon's Ministry of Public Health [64]	50.7% maximum in Beirut governorate [64]	Higher = worse
	Prenatal, Neonatal, and Maternal Deaths	Total still births, Intra-Uterine Fetal Demise (IUFD), neonatal deaths, and maternal deaths per 1,000 newborns from vital data observatory (VDO) statistics	Hospitals report VDO statistics to Lebanon's MoPH [65]	Not determined	Higher = worse
	Pre-Mature Births and Birth Defects	Pre-mature births and birth defects per 1,000 live births from VDO statistics	Hospitals report VDO statistics to Lebanon's MoPH [65]	Not determined	Higher = worse
	Total Mandatory Notifiable Diseases	Total vaccine preventable disease, food / water borne diseases, and other diseases per 100,000 people	Lebanon's MoPH surveillance data [66]	Not determined	Higher = worse
**D_2 — Capacity Strain (w_S)**	PHC Center Density	Number of PHC centers per 100,000 Lebanese and non-Lebanese people for both PHC center density as well as for total mandatory notifiable diseases above	Hemadeh et al. (2020) [12]	Minimum 2.951 PHC centers per 100,000 people in governorate of Mount Lebanon [12]	Lower = worse
	Staffing Adequacy Gap	PHC centers employing all clinical staff per national PHC standards (%)	Human Resources roster / staffing log (existing)	< 57.6% (national baseline) [12]	Lower = worse
**D_3 — Socio-economic Vulnera-bility (w_V)**	Displacement Share	Governorate population who are non-Lebanese (%)	Lebanon Ministry of Public Health statistical bulletins [50]	> 47% [12,16]	Higher = worse
	Food Insecurity	Household with food insecurity, including mild, moderate, and severe categories (%)	2023 survey by Lebanon's Ministry of Public Health (MoPH) [64]	Maximum observed of 93.5% in governorate of Akkar [64]	Higher = worse
